# Anti-Dopamine Receptor 2 Antibody-Positive Encephalitis in Adolescent

**DOI:** 10.3389/fneur.2020.00471

**Published:** 2020-06-16

**Authors:** Xuejiao Dai, Lilu Kuang, Li Feng, Xiaoping Yi, Weiting Tang, Qiao Liao, Xiaoyan Long, Junling Wang, Jing Li, Huan Yang, Bo Xiao, Guoliang Li, Si Chen

**Affiliations:** ^1^Department of Neurology, Xiangya Hospital, Central South University, Changsha, China; ^2^Department of Neurology, The First Affiliated Hospital, Zhejiang University, Hangzhou, China; ^3^Department of Neurology, Liuyang Jili Hospital, Changsha, China; ^4^Department of Radiology, Xiangya Hospital, Central South University, Changsha, China

**Keywords:** D2R, basal ganglia encephalitis, adolescent, movement disorder, psychiatric

## Abstract

Autoimmune encephalitic syndromes include mutism, somnolence, movement disorder, and behavioral, or psychiatric symptoms. When paired with bilateral basal ganglia lesions on magnetic resonance imaging, these support the diagnosis of basal ganglia encephalitis (BGE). BGE is a rare but distinct entity of putative autoimmune etiology, with specific basal ganglia inflammation and acute movement disorders. A previous study identified dopamine-2 receptors (D2R) antibody to be positive in most BGE children, indicating that the D2R antibody may trigger the downstream pathological process in BGE patients. The highest levels of D2R are found in the striatum, the nucleus accumbens, and the olfactory tubercle. D2R antibody-positive BGE is widely reported in children. Here we present a 17-year-old girl with a typical clinical feature of basal ganglia encephalitis, who benefited from immune therapy.

A previously healthy 17-year-old Chinese girl was admitted to our institution with psychological and behavioral abnormalities for 9 months. Before the 1st admission on September 17, 2018, she was noted to walk unstably and gradually had akinesia for 12 days. Vomiting and generalized body rash were presented after she took some painkillers due to neck and shoulder pain, followed by a gradual psychiatric disorder. Psychological and behavioral abnormalities were noted, including reduced speech and movement. She reported no fever, headache, dizziness, or disturbance of consciousness. Her medical history included chronic gastritis and dog bite. She is allergic to ceftazidime, and she denied alcohol or drugs abuse. The neurologic examination revealed reduced muscle strength (4-) of the lower limbs and increased muscle tone of the limbs. Tendon reflex was decreased, and there was no muscle atrophy or hypertrophy. The patient was unable to follow the commands of a coordination test. Previous routine hematological and blood chemistry findings were within the normal range except for a mild increase of homocysteine (67.62 μmol/L, normal range <15 μmol/L), total bilirubin (26.4 μmol/L, normal range <17.1 μmol/L), and IgG and IgA in the cerebrospinal fluid (CSF) (IgG: 0.09 g/L, normal range <0.03 g/L; IgA: 14.9 mg/L, normal range <11.1 mg/L). A slight decrease of ceruloplasmin (202.0 mg/L, normal range 260–360 mg/L) was also noted. The results of blood tests were negative for vasculitis, inflammation (virus and epidemic encephalitis B virus), and demyelinating or other metabolic diseases, and CSF testing was normal. Autoimmune encephalitis antibodies including NMDAR, AMPA1, AMPA2, LGI1, CASPR2, GABA B receptor, DPPX, IgLON5, and GAD65 and anti-neuron IgG including amphiphysin, CV2, PNMA2(Ma2/Ta), Ri, Yo, and Hu in the serum and in the CSF were negative.

The brain MRI revealed symmetric lesions in the bilateral basal ganglion with hyper-intensity on spin-echo T1-weighted images. Symmetric hypo-intensity was also found in the internal capsule. The T2-weighted image and diffusion-weighted imaging (DWI) results were normal (Figure 1). Gynecological ultrasonography revealed a smaller uterus and bilateral polycystic ovarian. The electroencephalogram showed an increased slow-wave present over the bilateral occipital and left frontotemporal regions. The electromyography and abdominal ultrasound results were unremarkable. A presumed diagnosis of autoimmune encephalitis was considered due to the representative clinical manifestations after the first admission to our institution, even though a specific antibody was not detected in the serum or the CSF. The patient was treated with a high dose of glucocorticoid (1,000 mg methylprednisolone sodium succinate, half of it every 3 days) and gradually shifted to oral prednisone taper. Intravenous immunoglobulin was also used. A recovery from the behavioral abnormalities and on movement was noted after the treatment.

**Figure 1:**
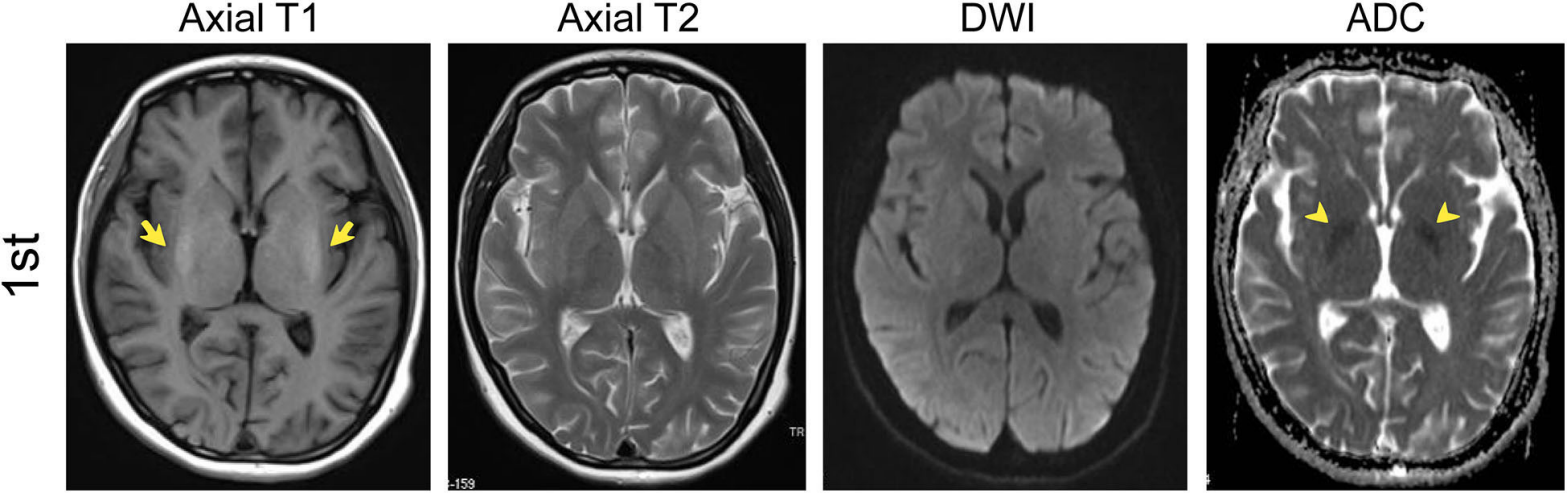
MRI results of the first admission (in September). T1, T2, DWI, and Apparent diffusion coefficient (ADC) axial images in September (1st) showed symmetric lesions in the bilateral basal ganglion with hyper-intensity (yellow arrows) and symmetric hypo-intensity of ADC in the internal capsule (yellow arrowheads).

However, during the process of low-dose oral prednisone, she presented with uncontrolled head fall backward for 1 month and was admitted to our institution for the 2nd time on January 8, 2019. The physical examination showed neck stiffness and fine tremor of limbs. Her CSF testing was normal, except for leucocyte at 4.0 × 10^6^/L. No antibody was detected in the CSF or the serum this time either. A follow-up MRI in January revealed symmetric iso-intensity in the bilateral basal ganglion on slightly enhanced T1-weighted images (Figure 2A). The bilateral basal ganglion with decreased signal intensity was noticed in apparent diffusion coefficient (ADC) images. The T2-weighted image was unremarkable.

**Figure 2:**
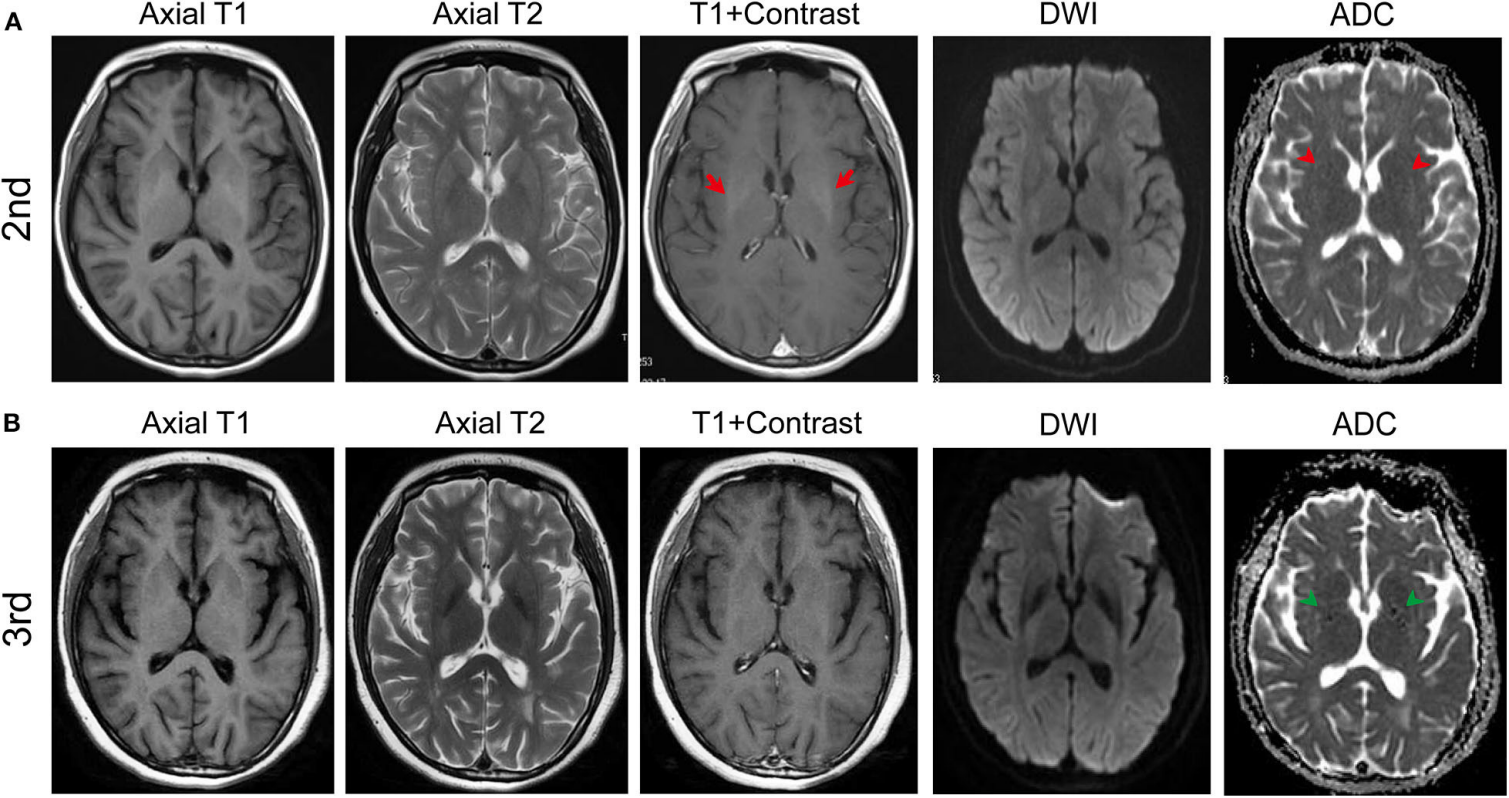
MRI results of the second (in January) and the third admission (in Jun). **(A)** T1, T2, enhanced T1, DWI, and apparent diffusion coefficient (ADC) axial images in January (2nd). After the immune therapy, the follow-up T1 axial image in January revealed symmetric iso-intensity but enhanced slightly (red arrows) in the bilateral basal ganglion. Dotted hypo-intensity of ADC in the internal capsule (red arrowheads) was noticed. **(B)** Follow-up MRI in June (3rd) showed the iso-intensity of the bilateral basal ganglion with no enhancement and the dotted hypo-intensity lesion in the internal capsule (green arrowheads).

The clinical manifestations of movement disorders, symmetric lesion in the bilateral basal ganglion, and a recovery in the MRI result within a period of time after the immune therapy suggested a diagnosis of basal ganglia encephalitis, although it is rare and mostly reported in pediatric cases. Hence, immune therapy was restarted concomitant with mycophenolate mofetil due to the new symptom, and serum anti-dopamine receptor 2 antibody was tested, which leads to a diagnosis of basal ganglia encephalitis as widely reported in mostly in children. Human D2R-Ab Elisa kit (Shuangying, China) was used to detect, and the titers of serum anti-D2RAb were increased (49.02 U/L, normal range 5–36 U/L), which revealed D2R-Ab-positive basal ganglia encephalitis.

On June 10, 2019, the patient was admitted to our hospital with a new symptom of limb trembling for 5 months. The follow-up MRI on June 13, 2019 showed the remaining decreased signal intensity in bilateral ganglia on T1-weight sequence (Figure 2B). Her CSF testing was normal except for increased anti-D2RAb (86.27 U/L). Also, the D2R antibody in the serum increased to 51.05 U/L compared to 49.02 U/L of D2R antibody during the 2nd admission. Trihexyphenidyl was used to control the tremor. Memory B cell was tested before the rituximab treatment. After the immune treatment, head fall backward was partly controlled with an unfortunate residual symptom of limb trembling.

## Discussion

Dopamine is a crucial neurotransmitter in the brain. The G protein-coupled seven-transmembrane dopamine receptors (D1, D2, D3, D4, and D5) mediate all the physiological functions of the catecholaminergic neurotransmitter dopamine, ranging from voluntary movement and reward to hormonal regulation and hypertension (1). Common human diseases, such as Parkinson's disease, Tourette's syndrome, and schizophrenia, are related to dopaminergic dysfunction (1). Five receptors have been identified in humans and are divided into two groups: D1-class (DRD1 and DRD5) and D2-class receptors (DRD2, DRD3, and DRD4) based on their structural, biochemical, and pharmacological properties (2, 3). The highest levels of D2 dopamine receptors are found in the striatum, the nucleus accumbens, and the olfactory tubercle. The substantia nigra, ventral tegmental area, hypothalamus, cortical areas, septum, amygdala, and hippocampus are also found to express D2 receptors (4–6). D2R is intimately linked to the control of movement and behavior, which could be a reason of movement and psychiatric disorders associated with D2R antibody. A recent study further pointed out that the dopamine-2 receptor extracellular N-terminus regulates receptor surface availability and is the target of human pathogenic antibodies from children with movement and psychiatric disorders (7).

Anti-D2R antibody-positive basal ganglia encephalitis is a type of autoimmune encephalitis, which is mostly reported in pediatric cases. A previous study compared the clinical feature of anti-D2R antibody-positive and -negative encephalitis in children. The symptom onset frequently occurred in the post-infectious or post-vaccine setting, with 1/12 of them occurring after medication. The symptoms at onset were variable, but movement disorders dominated the clinical syndrome of D2R antibody-positive encephalitis and included dystonia, dystonic tremor, oculogyric crises, parkinsonism, and chorea. The psychiatric features were common but not universal. Sleep disturbance was also common. Seizures occurred only in 20% of the D2R antibody-positive encephalitis. MRI was normal in 50% of the D2R antibody-positive encephalitis cases, but when abnormal, the lesions were localized to the basal ganglia (8). A previous study also suggested that an early treatment with intravenous immunoglobulin (IVIG) may be associated with better long-term neuropsychological outcomes since it reduces the risk of a relapse (9).

The first-line therapy for the acute disease is intravenous (IV) or oral pulse of methyl-prednisolone or equivalent, IVIG, and/or plasma exchange. The second-line therapy for the acute disease is rituximab or cyclophosphamide or both. Maintenance therapy is monthly IV or oral steroid pulses or oral prednisolone taper, monthly IVIG (3–12 months, depending on the severity and the course). In a refractory patient who has failed in the first- and second-line therapy, intrathecal methotrexate is considered, a monoclonal antibody against IL6 (tocilizumab). To treat the chronic disease and prevent relapse, azathioprine, mycophenolate, mofetil, and re-dosing rituximab could be used (10).

In this case, a 17-year-old girl presented with a typical clinical feature of basal ganglia encephalitis: movement disorder (dystonia, walking unstably and gradually resulting to akinesia, and fine tremor) and psychiatric features. MRI showed recurring symmetric lesions in the bilateral basal ganglion, with hyperintensity on T1-weighted images. The titers of anti-D2R antibody were found to be increased in the patient's serum and CSF. Immune therapy also benefited to her recovery. The treatment of our patient strictly followed the guideline, including the first-line, second-line, maintenance, and chronic disease therapy. Anti-D2R antibody-positive basal ganglia encephalitis is mostly reported in pediatric cases. Our case indicated the similar pathogenic process and the clinical manifestation of anti-D2R antibody-positive basal ganglia encephalitis in juveniles.

The patient exhibits unusual MRI features with T1 hyperintensity of the basal ganglia and with no alteration of T2. Her follow-up MRI in January revealed enhanced T1-weighted images (Figure 2A). The dynamic recovery in MRI after immune therapy may suggest recovery from basal ganglia encephalitis. Therefore, the abnormal MRI features in T1 and T1 enhancement of the basal ganglia were novel discoveries in the case. Considering that some cases of basal ganglia encephalitis occurred in the post-infectious or the post-vaccine setting, we think that it may be because of the manganese deposit or some subacute cerebral hemorrhage and edema in the basal ganglia. Also, D2R antibodies were determined using an ELISA without confirmation by cell-based assay (CBA) or tissue-based assay (TBA). The decreased concentration of the D2R antibody with ELISA tests after the immune therapy and the negative results of other autoimmune encephalitis antibodies, such as NMDAR, AMPA1, AMPA2, LGI1, CASPR2, GABA B receptor, DPPX, IgLON5, and GAD65, which were detected by indirect immunofluorescence (IIF; including CBA and TBA) and ELISA, may indicate the diagnosis. Future work should concentrate on the stable detection of D2R antibodies with IIF.

## Data Availability Statement

All datasets generated for this study are included in the article/supplementary material.

## Ethics Statement

Written informed consent was obtained from the individual(s), and minor(s)' legal guardian/next of kin, for the publication of any potentially identifiable images or data included in this article.

## Author Contributions

XD, LK, QL, and LF acquired the clinical data. XD drafted the manuscript. SC supervised the study and revised the manuscript. XY performed and analyzed the MRI. WT, XL, and JW revised the manuscript. JL, HY, BX, and GL polished the language.

## Conflict of Interest

The authors declare that the research was conducted in the absence of any commercial or financial relationships that could be construed as a potential conflict of interest.
